# Mobile Phone Apps for the Prevention of Unintended Pregnancy: A Systematic Review and Content Analysis

**DOI:** 10.2196/mhealth.4846

**Published:** 2016-01-19

**Authors:** Emily Rose Mangone, Victoria Lebrun, Kathryn E Muessig

**Affiliations:** ^1^ Gillings School of Global Public Health Department of Health Policy and Management UNC Chapel Hill Chapel Hill, NC United States; ^2^ Gillings School of Global Public Health Department of Health Behavior UNC Chapel Hill Chapel Hill, NC United States

**Keywords:** mHealth, eHealth, mobile phone, app, systematic review, unintended pregnancy, family planning, pregnancy prevention, contraception

## Abstract

**Background:**

Over 50% of pregnancies in the United States are unintended, meaning that the pregnancy is mistimed, unplanned, or unwanted. Unintended pregnancy increases health risks for mother and child, leads to high economic costs for society, and increases social disparities. Mobile phone ownership is rapidly increasing, providing opportunities to reach at-risk populations with reproductive health information and tailored unintended pregnancy prevention interventions through mobile phone apps. However, apps that offer support for unintended pregnancy prevention remain unevaluated.

**Objective:**

To identify, describe, and evaluate mobile phone apps that purport to help users prevent unintended pregnancy.

**Methods:**

We conducted an extensive search of the Apple iTunes and Android Google Play stores for apps that explicitly included or advertised pregnancy prevention or decision-making support in the context of fertility information/tracking, birth control reminders, contraceptive information, pregnancy decision-making, abortion information or counseling, sexual communication/negotiation, and pregnancy tests. We excluded apps that targeted medical professionals or that cost more than US $1.99. Eligible apps were downloaded and categorized by primary purpose. Data extraction was performed on a minimum of 143 attributes in 3 domains: (1) pregnancy prevention best practices, (2) contraceptive methods and clinical services, and (3) user interface. Apps were assigned points for their inclusion of features overall and for pregnancy prevention best practices and contraceptive information.

**Results:**

Our search identified 6805 app descriptions in iTunes and Google Play. Of these, 218 unique apps met inclusion criteria and were included in the review. Apps were grouped into 9 categories: fertility trackers (n=72), centers and resources (n=38), birth control reminders (n=35), general sexual and reproductive health (SRH) information (n=17), SRH information targeted specifically to young adults (YA) (n=16), contraceptive information (n=15), service or condom locators (n=12), pregnancy tests (n=10), and games (n=3). Twelve apps scored at least 50 points (out of 94) for overall number of features and at least 15 points (out of 21) for contraceptive information and pregnancy prevention best practices. Overall, 41% of apps did not mention any modern contraceptive methods and 23% mentioned only 1 method. Of apps that did mention a modern contraceptive method, fewer than 50% of these apps provided information on how to use it. YA SRH apps had the highest percentage of pregnancy prevention best practices in each app. Demographic and interface evaluation found that most apps (72%) did not target any race and only 10% explicitly targeted youth. Communication interface features were present in fewer than 50% of apps.

**Conclusions:**

This review identified several useful, evidence-based apps that support the prevention of unintended pregnancy. However, most apps miss opportunities to provide users with valuable information, interactive decision aids, and evidence-based interventions for unintended pregnancy prevention. Further, some apps in this space may increase the likelihood of unintended pregnancy due to the low effectiveness of the contraceptive methods promoted.

## Introduction

Over 50% of pregnancies in the United States are unintended [1]. An unintended pregnancy is a pregnancy that is mistimed, unplanned, or unwanted at the time of conception, as the result of interrelated factors associated with knowledge, access, and behavior [2]. Unintended pregnancy increases the likelihood of adverse maternal and child health outcomes and results in high economic costs; US society supports US $11 billion in annual public insurance costs for pregnancy and first-year infant care [2,3]. Unintended pregnancy disproportionately affects younger women 18-24 years old, African American women, and lower-income women, contributing to cycles of poverty and inequality in the United States [2,4,5].

For these reasons, reducing unintended pregnancy is a compelling and complex public health challenge and a national public health goal. The US Department of Health and Human Services’ *Healthy People 2020* campaign aims to reduce unintended pregnancy by 10% by 2020 [6]. One way that they are doing this is by supporting evidence-based family planning programs, including those that leverage smartphones to deliver reproductive health information [7-12]. However, most smartphone family planning research focuses on text-message based approaches. Because smartphones are a relatively novel mechanism of health information delivery and behavioral intervention, there is little evidence on how smartphone technology is being leveraged to prevent unintended pregnancy.

Mobile phone apps have rapidly expanded in scope, sophistication, and reach, presenting a unique opportunity to put tools for pregnancy prevention in the pockets of millions of Americans. More than 145 million people in the US (58% of the mobile market) have a smartphone and that number is projected to increase to 220 million by 2018 [13]. Additionally, smartphone ownership demographics align well with those at high risk for unintended pregnancy; 77% of low-income 18- to 29-year-olds own smartphones [14]. Although health-related apps proliferate, there is no evidence that evaluates how they are being used to prevent pregnancy. Our research team conducted a systematic review of smartphone apps to answer the following questions:

Q1. What types of apps are currently available for family planning and pregnancy prevention?

Q2. Through what mechanisms are these apps preventing unintended pregnancy and are these approaches evidence-based?

Q3. What features of mobile technology are included in the user interface of these apps?

Q4. Who are the intended users of these apps and what is their feedback on these apps?

## Methods

### Search and Screening Strategy

In September 2014, we developed a comprehensive set of 30 search terms by using Medical Subject Headings terms and by consulting with researchers leading 2 other ongoing reproductive health systematic reviews. We also developed the following set of inclusion and exclusion criteria:

1. App includes or advertises at least one component of pregnancy prevention or decision-making such as the following: fertility information (ie, charting, information, etc) that claims to help prevent pregnancy; birth control reminders that claim to help prevent pregnancy; contraception information that explicitly notes pregnancy prevention; pregnancy decision-making information; sexual communication or negotiation information focused on preventing pregnancy.

2. App provides abortion information or counseling.

3. App is a pregnancy test (not including practical joke pregnancy tests).

4. App is for personal use by individuals looking to make decisions about their pregnancy (ie, not for clinicians, researchers);

5. App is not specific to one event (such as a conference);

6. App is in English;

7. App does not cost more than US $1.99.

To be included in the review, the app had to either explicitly state that it could help prevent unintended pregnancy or have content that supported unintended pregnancy prevention or decision-making, such as information on how to negotiate safe sex, clarification of pregnancy intention, or information about birth control. We also included apps that provided abortion counseling or related information following the rationale that individuals seeking information about abortion may have significant need for improved reproductive health/family planning services to prevent future need for abortion. We therefore wanted to assess the information provided at this key juncture. Similarly, we included pregnancy test apps that may not have a primary purpose of preventing unintended pregnancy but may be the first point of access for individuals whose contraceptive method has failed and, regardless of whether they are pregnant, may be in need of improved family planning or contraceptive counseling.

We only included apps that cost US $1.99 or less because we wanted to evaluate apps that were at a price point that is accessible to the majority of consumers. While most apps were indeed free, we did not limit the review to free apps because we also wanted to assess whether there were significant quality differences between paid and unpaid apps. After a brief, preliminary review of apps, US $1.99 was selected as an appropriate cutoff given the high availability of apps at the US $0.00, US $0.99, and US $1.99 levels. Additionally, paid apps that advertised the same services as their free counterparts were excluded to avoid duplication.

Each search term was put into both the iTunes store and Google Play store search engines and the resulting number of app descriptions was recorded for each search term (see Appendix I for search terms and search engine results). Search terms with high returns, such as “sex,” were automatically capped at 500 app descriptions by iTunes and 250 app descriptions by Google Play. All app descriptions were screened for relevance based on the inclusion criteria; however, because of the high number of returned app descriptions and there being no way to export the app descriptions into a spreadsheet, only apps that met inclusion criteria were documented. Therefore at the app description review level, we were unable to document the individual reasons for the exclusion of 6183 apps. Among the 4 reviewers, inter-rater reliability (IRR) checks were done independently for 20% of app description results in iTunes and Google Play (40% total), with IRR ranging from 94% to 100%. An additional screening of 100 (33%) of the included app descriptions was done collaboratively by all 4 reviewers for further quality control and shared understanding.

### Codebook Development

The codebook and data extraction form were finalized using examples from ongoing systematic reviews, as well as sources of pregnancy prevention best practices [15-17]. We established 3 broad domains—(1) pregnancy prevention best practices, (2) contraceptive information and clinical services, and (3) user interface—and data extraction points were created in each domain. Additional purpose-specific questions were developed once the primary purpose categories for all apps were finalized. Purpose-specific questions were developed for the young adult sexual and reproductive health information apps, birth control reminders, and fertility tracking apps in order to capture the nuances of these particular pregnancy prevention strategies. In total, the codebook included 143 data extraction points for every app, plus up to an additional 15 purpose-specific data extraction points.

### Data Extraction and App Assessment

Data extraction occurred in November 2014. In this study, 2 researchers with iPhones were assigned to iTunes and 2 with Android phones to Google Play. Apps were downloaded to the respective phones and 10% of those apps were co-reviewed between same-platform reviewers, with IRR scores of 92% and 95%.

#### Q1. App Purpose

To answer the first research question about what types of apps are in the family planning space, apps were categorized by their perceived primary purpose (eg, birth control reminders, fertility trackers, etc). Categorization was done on an iterative basis as the app descriptions were reviewed in the stores and was finalized by the team during the data extraction phase when content was reviewed in depth. Apps were assigned only to 1 category.

#### Q2. Mechanism of Pregnancy Prevention and Evidence-Based Practices

We used 3 methods to evaluate the mechanisms through which the apps attempted to prevent unintended pregnancy and their use of evidence-based approaches.

After a thorough review of PubMed and the gray literature, we identified a recent evidence-based clinical practice guideline for the prevention of unintended pregnancy that incorporated interventions for pregnancy prevention and evidence from federal and non-governmental programs into a comprehensive framework for unintended pregnancy prevention [16]. These guidelines provide a full spectrum of essential primary, secondary, and tertiary prevention services, including:

1. *Primary:* Assessment of personal and family health risk factors for unintended pregnancy (including intimate partner violence and substance abuse), appropriate screening tests, and prevention services;
2. *Secondary:* Assessment of pregnancy status, options counseling, support to continue pregnancy, and early abortion care; and
3. *Tertiary:* Pregnancy diagnostics/screening, crisis counseling, options counseling, support and termination referral coordination.

We further distilled these guidelines into the 7 pregnancy prevention best practice queries below, which either directly prevent unintended pregnancy (P1, P2, P4, P5, P7) or potentially lead to support for the termination of a current pregnancy or the prevention of future unintended pregnancy (P3, P6). We assessed each app for the inclusion of these 7 items, assigning 1 point per included item for total possible score ranging between 0 and 7.

P1. Does the app screen for or provide information about substance abuse?

P2. Does the app screen for or provide information about intimate partner violence?

P3. Does the app refer the user to pregnancy testing?

P4. Does the app ask the user to consider a life plan or their pregnancy intentions?

P5. Does the app provide behavioral contraceptive counseling?

P6. Does the app provide abortion counseling or referral?

P7. Does the app provide information about emergency contraception?

Second, to summarize the overall inclusion of information about contraceptives, we assessed the presence or absence of a mention of 14 barrier, device, or drug-based/hormonal contraceptive methods (eg, male condom, female condom, diaphragm, sponge, cervical cap, oral contraceptive, injectable contraceptive, intrauterine device (IUD), implant, patch, ring, spermicide, emergency contraception, and surgical sterilization) from the World Health Organization’s modern methods list and the more comprehensive Planned Parenthood birth control list, as well as less effective methods such as fertility tracking and withdrawal [18-20]. We also assessed whether the app described how to use the contraceptive method, and whether the app provided information on the effectiveness of the contraceptive method. Finally, we evaluated whether the app provided information on where to access (any) contraceptives (eg, at a clinic, a pharmacy, a grocery store), whether the app located (any) contraceptives near the user, whether the app provided (any) information on contraceptive risks or side effects, whether the app included (any) information on side effect management or switching contraceptive methods, and whether the app discussed dual protection (ie, prevention of both pregnancy and sexually transmitted infections (STIs)).

Third, we quantitatively scored each app using 2 scores: (1) the first based on the total number of desirable features (interface and content) included out of 84 possible features and (2) the second score based on the number of contraceptive methods out of the 14 noted above and the number of 7 possible pregnancy prevention best practices included in the app (possible score of 21; see Appendix III). We qualitatively described any innovative or useful features in the top-scoring apps. If the app provided a clear overall message about how to avoid unintended pregnancy, it was weighted to receive 10 points. For example, to meet this criteria fertility trackers had to go beyond notifications like “you are fertile today” and provide actionable prevention guidance such as “use a condom during sex to prevent pregnancy this week.”

#### Q3. User Interface

Answering the third research question about which features of smartphone technology were represented was done with consultation from an app development specialist who helped clarify and define the different components of smartphone user interface. We reviewed apps for 16 desirable interface features, 13 of which could directly support prevention of unintended pregnancy through access, knowledge, or communication, such as GPS, maps navigation, clinic/service locators, contraceptive locators, interactivity (ie, setting profiles and getting tailored information), appointment scheduling, public/forum communication, direct communication (eg, chat, text, call), push notifications, informative videos, audio, and a decision aid feature to help users actualize pregnancy intentions, clarify contraceptive preferences, and/or make decisions about sexual partners or activity. An additional 3 positive features, including customizable skins/background, main menu/navigation bar, and text/image clarity, were included in the evaluation because they contribute to user experience and the likelihood that a user will continue to use the app or recommend it to peers. We also evaluated apps for the presence of 4 undesirable components of interface, including whether the app crashed during review, whether navigational links were broken, whether there was advertising, and whether the app required purchase after being downloaded in order to use the basic features advertised in the free version.

#### Q4. Targeted Users and User Feedback

Demographic information about app users was not publicly available, so we devised a methodology to answer this research question based on the images in the apps. To evaluate racial inclusion, we reviewed all of the images of people in the apps and classified each image as Caucasian, Hispanic, African American, East Asian and/or Indian based on physical appearance. When there was racial or ethnic ambiguity, the image was tagged in multiple race/ethnicity categories.

We also reviewed for gender-targeting. Apps were deemed to be targeting females if it was explicitly stated or if the apps only provided services that are relevant to a female (such as tracking her own menstrual cycle). Apps were deemed to be targeting males if the app only provided male sexual or reproductive health information. Apps that were categorized as gender-inclusive were apps for shared tracking of fertility or birth control information, gender-neutral service/contraceptive locators, or gender-neutral or gender-inclusive information about contraceptives and sexual and reproductive health (SRH). In terms of age, we reviewed for general versus young adult targeted apps. Apps were categorized as young adult if the app title or description stated that it was for “teens,” “young adults,” “girls,” “boys,” “youth,” “adolescents,” or “students.”

Finally, we conducted an overall analysis on user ratings and downloads. iTunes does not provide information on number of downloads (an indication of app popularity) so only Google Play apps were analyzed for use and ratings.

## Results

### Search Results and Categories

**Figure 1:**
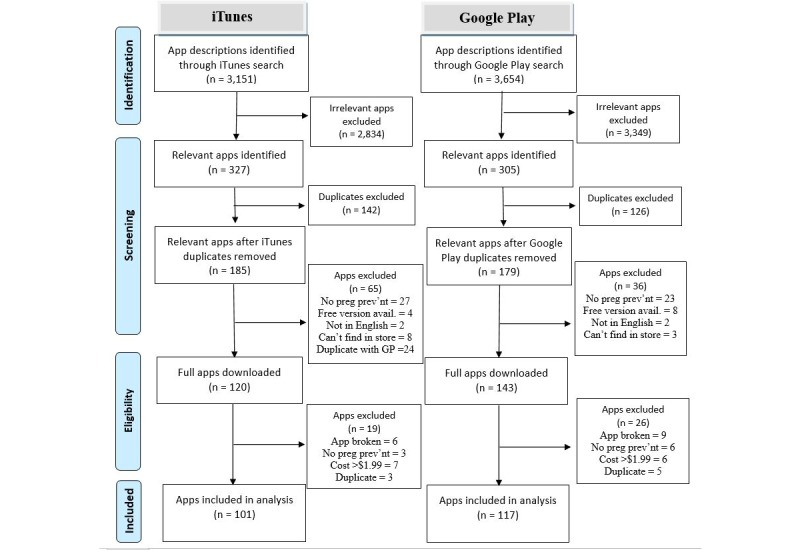
PRISMA diagram for iTunes and Google Play search results.

Our search strategy resulted in 6805 app descriptions in iTunes (3151) and Google Play (3654) (see Figure 1 for a PRISMA diagram of the search results [21]). Of these, 218 unique apps met inclusion criteria and were included in the review (101 in iTunes, 93 in Google Play, and 24 listed in both). See Appendix II for full list of included apps. Apps that were available in both iTunes and Google Play were removed from the iTunes group and only analyzed as Google Play apps to avoid duplication. Within the iTunes and Google Play stores, 105 included apps were found in the health and fitness (48%), 68 apps in medical (31%), 20 in lifestyle (9%), 12 in education (6%), and 13 in other store departments (6%).

#### Q1. App Purpose

**Figure 2:**
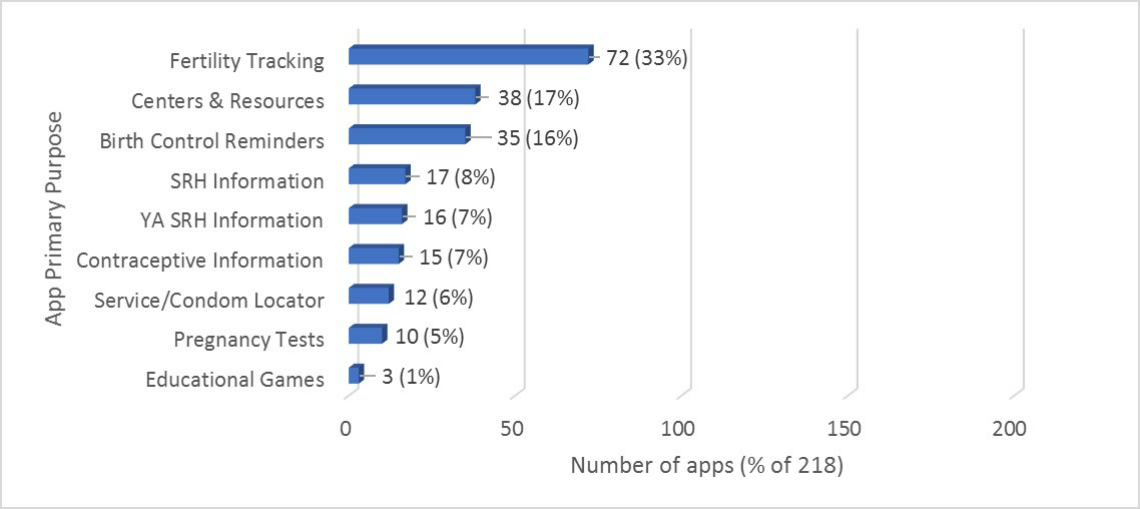
Primary purposes of 218 apps that support unintended pregnancy prevention.

Nine “primary purpose” categories were generated based on the apparent purpose and content of the 218 included apps (Figure 2). These categories were fertility tracking apps (n=72), centers and resources (n=38), birth control reminders (n=35), SRH information apps (n=17), SRH apps for young adults (YA) (n=16), contraceptive information apps (n=15), contraceptives or service locator apps (n=12), pregnancy tests (n=10), and education games (n=3). The largest category, fertility trackers, included apps that explicitly advertised that they could help women prevent pregnancy by assisting the user with fertility awareness-based methods of family planning, also called natural family planning. Fertility trackers mostly utilized user-provided information about menstrual cycles and cervical biomarkers to predict when a woman would or would not be fertile. It is important to note that fertility tracking is more commonly used by those trying to become pregnant and these apps could be used for that purpose as well. No fertility tracking apps we identified were exclusively designed for the purpose of preventing pregnancy.

The next largest group of apps, centers and resources, was developed for facilities that offer clinical services, pregnancy testing, and/or abortion counseling. With a few exceptions, such as the Planned Parenthood app, these center-based apps primarily referred users to pregnancy support centers that provide pro-life or “life affirming” pregnancy options counseling. The third largest category, birth control reminders, explicitly advertised pregnancy prevention. Birth control reminder apps generally used visual cues such as blister packs or calendars, push reminders, and alarm features to remind the user to take their birth control. Birth control methods supported by these reminders were mostly daily oral contraceptives, but a few were for alternative contraception, including the ring (NuvaRing), the patch, and the shot (Depo-Provera). Other categories included apps that provided general SRH information and apps that provided SRH information in a more targeted manner to young adults. We also identified groups of apps that primarily provided contraceptive method information, typically as a descriptive list of types of contraceptives; apps that were for the purpose of locating SRH services or condoms using GPS features; pregnancy tests, which collected information about biological and behavioral user experience; and educational games.

#### Q2. Methods for Pregnancy Prevention

**Figure 3:**
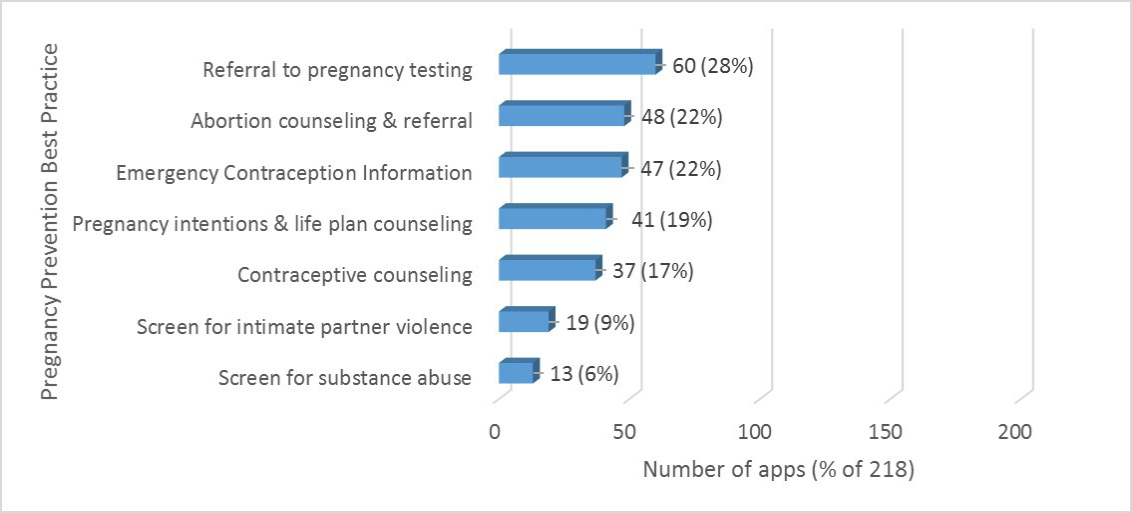
Pregnancy Prevention Best Practices Found in Apps.

Only 65 apps (30%) conveyed a clear message on how to prevent pregnancy. Of the 7 evidence-based best practices, screening for unintended pregnancy risk factors—such as substance abuse or intimate partner violence—were the least common practices in these apps, in 13 (6%) and 19 (9%) of apps, respectively (see Figure 3). Behavioral contraceptive counseling, defined as persuasive counseling that recommends or describes the importance of contraceptives, was also infrequently included. The most common evidence-based pregnancy prevention method in apps—referral to pregnancy testing—was offered by less than a third of all apps.

When we analyzed the presence of best-practices by app primary purpose, we found that YA SRH apps integrated the highest percentage of overall best practices into their services offered, followed by center and resource apps and general SRH information apps (Table 1). Fertility tracking apps, birth control reminder apps, and pregnancy test apps incorporated the fewest best practices for preventing unintended pregnancy.

**Table 1:** Number and percentage of pregnancy prevention best practices in apps by app purpose.

App primary purpose (n)	Pregnancy prevention best practices, n (%)							
	P1	P2	P3	P4	P5	P6	P7	3+^a^
Fertility Tracking (72)	0 (0)	0 (0)	2 (3)	1 (1)	6 (8)	0 (0)	1 (1)	0 (0)
Centers and Resources (38)	1 (3)	3 (8)	34 (89)	29 (76)	3 (8)	32 (84)	14 (37)	29 (76)
Birth Control Reminders (35)	0 (0)	0 (0)	2 (6)	0 (0)	1 (3)	0 (0)	0 (0)	0 (0)
Contraceptive Information (17)	1 (6)	0 (0)	2 (12)	3 (18)	5 (29)	1 (6)	8 (47)	3 (18)
SRH Information (16)	4 (25)	7 (44)	4 (25)	4 (25)	7 (44)	4 (25)	8 (50)	8 (50)
YA SRH Information (15)	7 (47)	8 (53)	9 (60)	3 (20)	11 (73)	9 (60)	11 (73)	10 (67)
Service/Condom Locator (12)	0 (0)	1 (8)	2 (17)	1 (8)	3 (25)	2 (17)	4 (33)	3 (25)
Pregnancy Tests (10)	0 (0)	0 (0)	5 (50)	0 (0)	0 (0)	0 (0)	0 (0)	0 (0)
Educational Games (3)	0 (0)	0 (0)	0 (0)	0 (0)	1 (33)	0 (0)	1 (33)	0 (0)

^a^3+ Includes 3 or more of best practices P1-P7.

### Contraceptive Information

**Figure 4:**
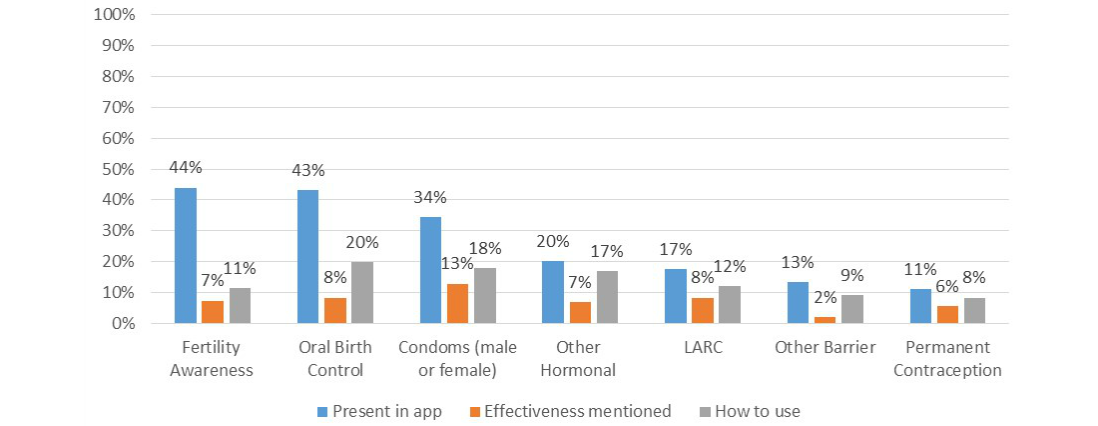
Percentage of mobile phone apps containing information about types of contraceptive methods, their effectiveness, and use.

Although there was a wide range of contraceptive methods that appeared in some apps, 89 apps (41%) did not mention any of the 14 modern contraceptive methods we evaluated and 51 apps (23%) mentioned only 1 of these methods. The most commonly mentioned methods were fertility awareness (96; 44%), oral birth control pills (94; 43%), and male or female condoms (75; 34%) (Figure 4). Of the apps that did mention a contraceptive method, less than half of all apps provided further information on how to use the method or the method’s effectiveness at preventing pregnancy.

SRH information, YA SRH information, and contraceptive information app categories had the greatest number of individual apps that mentioned the largest number of contraceptive methods. On average, apps in each of these 3 categories mentioned 7 contraceptive methods and were also more likely to mention the effectiveness of the contraceptive methods, compared with other app categories. Fertility tracking apps seldom mentioned any other method than fertility awareness except when discouraging the use of hormonal contraceptives because they affect the user’s menstrual cycle, making it harder to track. Birth control reminders rarely mentioned the use of contraceptive methods other than the particular method that app was promoting, even if the user entered data showing poor adherence.

In terms of access to contraceptives, 36 apps (17%) provided information on where users could access contraceptives (eg, clinics, pharmacies, grocery stores, etc) and 17 apps (8%) helped users locate contraceptives near them (using GPS or a search for that city). Unsurprisingly, contraceptive locator apps had the highest percentage of apps with access information, with 92% (11/12) of contraceptive locator apps including both where to seek contraceptives and help in locating these venues. YA SRH apps followed with 75% of YA SRH apps providing information on where a user could access contraceptives and 31% helped users locate specific points of sale. A total of 41% and 40% of SRH information apps and contraceptive information apps, respectively, provided information on where users could locate contraceptives—but only 1 app in each category provided specific locator services.

Contraceptive information apps, SRH information apps, and YA SRH apps had the highest percentage of apps that discussed contraceptive side effects (53%, 47%, and 44%, respectively), as well as the highest percentage of apps that discussed side effect management or switching methods (20%, 18%, and 13%, respectively). Notably, only 1 of 35 birth control reminder apps included any information on contraceptive side effects or switching. YA SRH apps, SRH information apps, and contraceptive information apps had the highest percentage of apps that included information about dual protection (ie, protection against both pregnancy and STIs) at 75%, 71%, and 53%, respectively.

### Scoring and Qualitative Evaluation

There was a strong correlation (0.93) between overall scores (out of 94) and contraceptive information/best practices scores (out of 21). Fifteen apps scored at least 50 points for overall features and at least 15 points for contraceptive information and pregnancy prevention best practices, but 3 were excluded from the “high-score” list because they crashed during testing and were therefore not reviewed here (Table 2). Of the 6 top-scoring apps, 1 app was on YA SRH information, 4 were general SRH information, 1 app was from the contraceptive information category, and 1 from the centers and resources category. Most of these apps had credible public health-related developers such as Planned Parenthood, New York City (NYC) Department of Public Health, and the National Health Service (NHS) in the United Kingdom (UK). In addition to the contraceptive information and pregnancy prevention best practices features, these apps included features like GPS, push notifications, decision aids, a clear description of how to avoid unintended pregnancy, information on dual protection and STIs, and many other of the 84 attributes that contribute to user experience and support the prevention of unintended pregnancy. In this study, 5 of the 11 top-scoring apps were developed in the United States and 4 were developed in the United Kingdom.

Two of the top-scoring apps, Mayo Clinic About Birth Control and SafeSex Guide, cost money to download (US $1.99 each). However, when comparing apps that were free (n=176) to paid apps (n=42), paid apps actually scored lower on average for both best practices (3.2 vs 3.8) and overall (13.4 vs 16.4).

In addition to the quantitative scoring, reviewers also wrote brief descriptions of the apps, including notable features or problems. Among the highest-scoring apps, there were several features that are worth highlighting. The *Mayo Clinic: About Birth Control* app included a useful interactive decision aid that guided users to a contraceptive method that was a good fit for them based on a series of questions about pregnancy intentions, health conditions, personal habits, privacy requirements, and general preferences. This app also provided helpful videos, pictures, and a glossary of contraceptive information. A useful feature in the *My Sex Doctor Lite* app is a series of questions that lets young adults explore whether they are in an abusive or controlling relationship. The *My Sex Doctor Lite* app is structured in a question-and-answer format and also asks (and answers) other questions about sexual health, relationships, STIs, and unintended pregnancy. *Teens in NYC* navigates young adults through questions about where to go for sexual health services, what types of birth control are available, and what to expect at the clinic. A notable feature in the *Teens in NYC* app was its statement that “Teens in NYC have the right to sexual health services without getting permission from parents, girlfriends/boyfriends or anyone else,” empowering teens and addressing concerns about parental and partner consent as barriers to access and use. Finally, there was an interesting feature in the *C&SH Summerset* app called a “c-card” that allows users to get information about condoms, set their condom preferences, and use the app to “purchase” condoms for free from select retailers. Users can get their c-card key fob and app PIN from a community-based “Issuer” who provides counseling and other assistance. The top scoring apps used varying approaches but provided evidence-based features and services for pregnancy prevention.

#### Q3. User Interface and App Features

Table 3 provides key statistics on different features of the apps ranging from whether they had a main navigation menu (157; 72%), were interactive (ie, user had the ability to set personal preferences and input profile information in a way that the app provided tailored feedback) (138; 63%), used GPS (60; 28%), or facilitated communication. Noted communication features included push notifications from app to user (90; 41%), direct or public forum communication (28% and 27%, respectively), and appointment scheduling (25; 11%). We also evaluated whether the app allowed the user to personalize the appearance of the app (39; 18%), whether the app included video (25; 11%) or audio (13; 6%), or whether the app functioned as a sexual decision aid (ie, provided tailored recommendations or scenarios to support sexual decision-making) (17; 8%). Finally, we noted when there was advertising (69; 32%) and when there were faulty elements such as unclear images, unresponsive navigation, or app crashes (43; 20%).

**Table 2:** Highest scoring apps overall and for contraceptive/best practices.

App name	Developer/sponsor (country)	App category (primary purpose)	Platform	Contraceptives + best practices score (out of 21)	Overall score (out of 94)
Planned Parenthood Care	Planned Parenthood Federation of America (US)	Centers & Resources	Google Play & iTunes	21	69
Sexual Health Guide	GIRT Mobile (Ireland)	SRH Information	Google Play & iTunes	19	65
No Worries	Wiltshire College, Terrence Higgins Trust, and Salisbury PCT (UK)	SRH Information	Google Play	18	71
Your Choice Your Voice	NHS and Bromley Healthcare (UK)	YA SRH Information	iTunes	18	69
My Sex Doctor Lite	MYSD LTD; NHS (UK)	YA SRH Information	Google Play	18	63
C&SH Somerset	NHS (UK)	YA SRH Information	iTunes	18	58
Mayo Clinic About Birth Control	Mayo Clinic (US)	Contraceptive Information	iTunes	17	59
Girls Incorporated of Lynn	MDPH Office of Adolescent Health and Youth Development (US)	YA SRH Information	Google Play	17	54
SafeSex101	Associated Students UCLA (US)	YA SRH Information	iTunes	15	58
SAFE	Amphibia (Malaysia)	SRH Information	Google Play	15	55
SafeSex Guide	Mobile Identity Danmark ApS (Unknown)	SRH Information	iTunes	15	54
Teens in NYC	NYC Department of Health and Mental Hygiene (US)	YA SRH Information	Google Play & iTunes	15	53

**Table 3:** Key user interface features.

User interface features	Number of apps	%
Main navigation menu	157	72
Interactive	138	63
GPS	60	28
Push notifications	90	41
Direct communication (live chat or email)	62	28
Public communication (forums)	58	27
Appointment scheduling	25	11
Customizable look (skins, etc)	39	18
Video	25	11
Audio	13	6
Sexual decision aid	17	8
Undesirable: advertising	69	32
Undesirable: faulty element or app crashes	43	20

#### Q4. Target User Demographics

Most apps (158; 72%) did not include pictures from which target race could be discerned. When images were included in the apps they depicted Caucasians in 59 apps (27%), African Americans in 24 apps (11%), Asians in 18 apps (8%), Hispanics in 13 apps (6%), and Indians in 5 apps (2%). As much as 12% of apps contained images of Caucasians only while 15% included images of multiple races. The majority of apps (73%) had no images of people from which to determine race. As mcuh as 22% of apps were available in at least one language other than English.

A total of 123 apps (56%) targeted females, 5 (2%) targeted males, and 90 (41%) targeted both females and males. Apps for birth control reminding, fertility tracking, and pregnancy testing mostly targeted females (94%, 93%, and 90% of apps, respectively). All other app categories were mostly gender-inclusive. Only in the contraceptive information category did the number of apps targeting males outnumber the apps targeting females, and this was due to 3 male condom preference/male condom sizing apps.

A total of 21 apps (10%) explicitly targeted “youth,” “teens,” or “young adults” but (using the rating criteria in iTunes and Google Play) only 13% of apps were rated “high maturity” in Google Play or “17+” in iTunes, indicating that 87% of apps were considered appropriate for, though not necessarily tailored to, young adults.

In terms of use and feedback, fertility tracking apps were by far the most popular apps with total downloads at over 68 million and a per-app average of 1.68 million downloads (Table 4). In addition to being popular, fertility tracking apps were also relatively well-liked with an average rating of 4.06 out of 5 stars. Birth control reminders and pregnancy tests also had high numbers of total downloads and per-app downloads but less so than the fertility tracking apps. Educational games and apps for centers and resources were the least downloaded apps. Notably, while young adult sexual and reproductive health apps had relatively few downloads, they were the highest rated as a group with 4.37 stars, indicating user satisfaction with the services offered. Some purpose categories, such as centers and resources and YA SRH information, have more location-based services, which may account for the lower number of downloads and reviews.

**Table 4:** App use and rating information from Google Play apps^a^.

App purpose (n)	Total downloads (average of range)	Average downloads/app	Average rating (out of 5 stars)	Total reviews
Fertility tracking (41)	68,844,675	1,679,138	4.06	1,579,017
Birth control reminders (22)	1,835,550	83,434	3.76	40,082
Pregnancy tests (7)	1,006,050	143,721	3.21	3886
SRH information (10)	114,960	11,496	3.63	383
Contraceptive information (9)	101,259	11,251	3.93	681
Service/condom locator (5)	64,050	12,810	3.46	100
YA SRH information (11)	19,590	1781	4.37	151
Centers and resources (11)	1140	104	5.00	5
Educational games (1)	30	30	n/a	n/a

^a^App usage information was not available in iTunes Store.

## Discussion

### Principal Findings

This systematic review of commercially available apps for iPhone and Android phones found a limited number of credible, evidence-based family planning and pregnancy prevention apps. Furthermore, identifying these apps using the search tools available in the iTunes and Google Play stores was challenging and time-consuming. Our team had to review thousands of app descriptions because there was not a sensitive search tool offered by either store. Another challenge was that once relevant apps were identified, it was often difficult to determine who had created the app and whether it was a credible source of family planning information. For example, the search term “abortion” resulted in the inclusion of several pregnancy testing centers that provided “abortion counseling.” However, when our research team called the centers to learn more about them, we found that they were mostly “life-affirming” organizations that did not actually refer for abortion. The implications of these findings are that users may be overwhelmed with irrelevant and uninformative apps and not be able to find apps that are responsive to their health needs or personal choices regarding family planning and pregnancy prevention.

The most important takeaway from this review is that an in-depth review of app content revealed the frequent absence of evidence-based best practices for pregnancy prevention as well as substantive information about effective methods of contraception. Only 17% of apps mentioned long-acting reversible contraceptives, including the IUD and implant, which are the most effective and longest-lasting reversible methods for preventing pregnancy. Providing information about these methods, especially to high-risk adolescents and young adults, is critically important given the low levels of awareness among young adults and in the general population [22]. Also notable was that only about a third of apps mentioned condoms, which are the only method of contraception that protects against both unintended pregnancy and STIs. Oral contraceptives were the most commonly referred to method among all apps, but this was due to the high number of apps that had the specific purpose of reminding users to take birth control. Disappointingly, birth control reminders largely missed out on opportunities to provide additional information on what to do if a pill was missed, what to expect from side effects, and alternative or supplemental contraceptive methods.

We also expected much higher percentages of information about emergency contraception and abortion in pregnancy testing apps, and contraceptive counseling and emergency contraception information in apps that educate the user about contraceptive methods. Pregnancy test apps were uninformative and contraceptive information apps mostly provided lists of information rather than offer tailoring, contraceptive preference clarification, or screening services. Finally, we found an unfortunate lack of apps that helped users actualize pregnancy intentions and to clarify sexual and reproductive health decisions and contraceptive preferences.

While there were a few apps that were innovative, interactive, and evidence-based, most apps in this space missed opportunities to provide useful information or interventions for unintended pregnancy prevention. Sexual and reproductive health information apps, centers and resources, and young adult sexual and reproductive health apps had the highest inclusion of evidence-based pregnancy prevention practices and the top-scoring apps were mostly found in these categories.

Another important finding of this study was that the largest group of apps that explicitly advertises pregnancy prevention is fertility trackers that support natural family planning or fertility awareness methods. This is concerning because according to the US Centers for Disease Control and Prevention, fertility awareness methods are the least effective method of birth control, with a failure rate of 24% per year [20]. One caveat, noted earlier, is that all of the fertility tracking apps included in this review could also be used to try to become pregnant and it is not possible to discern the app user’s pregnancy intentions. However, with such a high number of downloads and high user approval rating, it is possible that users who are happy with the pregnancy promotion features of these apps may also use them for child spacing and pregnancy prevention purposes. However, if this group of users rely exclusively on fertility tracking apps for pregnancy prevention, it could lead to a high number of unintended pregnancies.

Centers and resources apps were not significantly different from center Web pages and included few features other than information about the centers (with the notable exception of the Planned Parenthood app). These apps therefore were not very useful as stand-alone pregnancy prevention interventions. Similarly, birth control reminders were narrowly focused on providing 1 service such as an alarm or push notification for reminders. Nearly 2 million women downloaded apps whose only pregnancy prevention feature was a daily reminder. These apps therefore largely missed an important opportunity to provide information about how to use the methods, the importance of staying on schedule, what to expect from side effects, and what to do if a dose was missed. These apps could have also been improved with the inclusion of information on dual protection, other contraceptive method options, and how to discuss contraceptive use with partners/parents/health providers.

It was also noteworthy to find that higher app price was not necessarily associated with higher app quality. Most of the 42 paid apps were for fertility tracking (n=19) and birth control reminding (n=13), which provided specific services. Only 2 of the top-scoring apps cost money, indicating that quality can be found—indeed may even be more likely to be found—in apps that are free to users because they are for the purpose of health promotion, rather than financial gain.

A final remarkable finding was the extremely small number of games that we were able to include in the review from the extraordinarily large number of games that appeared in the search results. What is worth noting is why the games were excluded, namely: games were almost exclusively played from the perspective of the “hero” sperm and the goal of most games was to avoid as many birth controls as possible in order to get to the egg and “win.” Most games were therefore excluded because they did not include component of pregnancy prevention.

### Limitations

This review is subject to several limitations. One challenge stems from the ineffective search tools available in the iTunes and Google Play stores, which limited our ability to accurately and easily identify relevant apps. Also, because iTunes and Google Play stores capped the app results at 500 and 250, respectively, we may have missed apps that would have been included if we had been able to find them.

A limitation noted earlier in this paper is that there is no way to distinguish who is using the apps or for what purpose. This is an especially important consideration for the fertility apps, which constitute the largest group of apps in this review but may be used primarily by women who are trying to become pregnant rather than those who are seeking to prevent pregnancy.

A third limitation is that because of the novelty of the app as a platform for supporting unintended pregnancy prevention, there is not an established framework or pregnancy prevention best practices specifically for mobile phone apps. We adapted an evidence-based clinical guideline for prevention interventions but acknowledge that these guidelines may not be relevant for every app evaluated. Along these lines, because our review was so large, many of our data extraction points were simplified to a simple presence or absence of a feature, rather than a review of the quality of that feature. As this field matures, the research presented here can help inform future work on establishing guidelines and quality frameworks for evidence-based approaches to using apps for the prevention of unintended pregnancy.

### Conclusions

The conclusion of this review is that while there are several innovative, interactive, and evidence-based apps that have credible developers and provide useful information or interventions to prevent pregnancy, these apps are difficult to identify because the large majority of apps miss opportunities to help users prevent pregnancy by providing effective information, interventions, or referrals. Even more concerning is the possibility that the use of some of these apps may lead to additional unintended pregnancies due to the ineffective methods promoted or the lack of comprehensive information. A more concerted effort to promote or at least distinguish apps with credible sources and evidence-based best practices is needed. While it can be challenging to identify and adhere to guidelines for best practices, app developers should be cognizant that guidelines exist and should attempt to include additional evidence-based best practices for pregnancy prevention in their apps. Additional research on the impact of these apps on user experience, knowledge, attitudes, skills, behaviors, and outcomes would provide helpful insight into the value and effectiveness of these apps.

## Appendix

Multimedia Appendix 1 I. Search Terms and Search Engine Results & Sensitivity II. App Names, Platforms, Developers, & Primary Purposes for 218 Included Apps III. Grading Criteria for Contraceptives and Best Practices and Overall Scores.

## Abbreviations

IRR: inter-rater reliability
IUD: intrauterine device
STI: sexually transmitted infection
SRH: sexual and reproductive health
YA: young adult
