# Supplementary material for: Mobile Phone Apps for the Prevention of Unintended Pregnancy: A Systematic Review and Content Analysis
Source: JMIR Mhealth Uhealth. 2016 Jan 19;4(1):e6. doi: 10.2196/mhealth.4846 (PMC4738182; doi:10.2196/mhealth.4846)
Supplement: Multimedia Appendix 1 [file mhealth_v4i1e6_app1.pdf]

Supplementary Appendix

I. Search Terms and Search Engine Results & Sensitivity

II. App Names, Platforms, Developers, & Primary Purposes for 218 Included Apps

III. Grading Criteria for Contraceptives and Best Practices and Overall Scores

I. **Search Terms and Search Engine Results & Sensitivity***

**Search Term**	**iTunes Hits**	**iTunes Relevant Apps**	**iTunes Sensitivity**	**Google Play Hits**	**Google Play Relevant Apps**	**Google Play Sensitivity**
Sex	500	6	1%	250	7	3%
"The pill"	500	24	5%	105	15	14%
Pregnant	482	25	5%	250	9	4%
Fertility	342	29	8%	250	29	12%
Plan B	296	1	0%	250	1	0%
"Family planning"	77	12	16%	74	19	26%
"Birth control"	71	50	70%	129	46	36%
Condom	70	39	56%	135	22	16%
Contraception	66	34	52%	164	45	27%
Abortion	59	47	80%	250	12	5%
Sperm	63	7	11%	231	2	1%
Reproductive	43	1	2%	250	5	2%
Sterilization	37	0	0%	101	0	0%
Depo	30	2	7%	250	0	0%
Contraceptive	27	17	63%	250	72	29%
"Morning after"	9	1	11%	90	2	2%
Abstinence	7	3	43%	119	5	4%
LARC	6	1	17%	250	0	0%
IUD	6	4	67%	36	1	3%
Uterine	6	0	0%	81	0	0%
"Planned Parenthood"	6	1	17%	8	1	13%
Tubal	5	1	20%	11	1	9%
Nuvaring	2	2	100%	18	10	56%
"Sexual communication"	1	0	0%	1	1	100%
"Sexual negotiation"	0	0	0%	0	0	0%
"Pregnancy prevention"	7	2	29%	4	3	75%
"Prevent pregnancy"	4	4	100%	10	7	70%
"Prevent conception"	1	1	100%	0	0	0%
"Sex education"	424	12	3%	59	8	14%
"Avoid pregnancy"	14	1	**7%**	28	14	50%
**Total**	**3161**	**327**	**10%**	**3654**	**305**	**8%**

**Sensitivity is the number of relevant apps divided by number of key word search hits*

II. **218 Included Apps**

Database	App Name	Seller/Developer	Primary Purpose
iTunes	Ring timer for iPhone	Raphael Bartolome	Birth Control Reminder
iTunes	D-Calc	Mark Ziemer	Birth Control Reminder
GP	Pill	dferreira	Birth Control Reminder
GP	Birth Control Pill Alarm	mamuso	Birth Control Reminder
iTunes	Don’t Forget The Pill	David Tessitore	Birth Control Reminder
iTunes	My Reminder - Birth Control Pill	Aspyre Solutions Pty Ltd	Birth Control Reminder
iTunes	MyBirthController	Alex Benyukhis	Birth Control Reminder
iTunes	Pilly! - Your Pill Reminder	Orcun Yoruk	Birth Control Reminder
iTunes	iPill	EH Partners SA	Birth Control Reminder
iTunes	Pink Reminder	Deep Pocket Series LLC	Birth Control Reminder
iTunes	RingRemind	Alena Kazarova	Birth Control Reminder
GP	Menstrual Calendar	EZHLD Corporation	Birth Control Reminder
iTunes	My OC Diary	Toroku Shimizu	Birth Control Reminder
iTunes	Simplest Pill Reminder	Luca Rullo	Birth Control Reminder
GP	contraceptive patch	JFMA	Birth Control Reminder
GP	Lady Pill Reminder	Sergio Viudes	Birth Control Reminder
GP	Contraceptive Pill Reminder	TD Incorporated	Birth Control Reminder
GP	Contraceptive Pill	JFMA	Birth Control Reminder
GP	Contraceptive ring	JFMA	Birth Control Reminder
Both	myPill BC Reminder	Bouqt	Birth Control Reminder
GP	Contraceptive ring HD	pm.app.software	Birth Control Reminder
iTunes	Period Tracker with Mood, Fertility & Birth Control Daily Diary	Rebellion Media	Birth Control Reminder
GP	LadysCalendar Pill Free	FalleryApp	Birth Control Reminder
GP	Ladies' Diary	PharmaPromo	Birth Control Reminder

GP	Lucky Lookout	Justin T Robinson Software	Birth Control Reminder
GP	Pill Reminder Lite	bropatapps	Birth Control Reminder
Both	My OC	Consilient Health Ltd	Birth Control Reminder
GP	Pilly!	Nucro	Birth Control Reminder
Both	BonaPill	ALFA MEDIMEDIA	Birth Control Reminder
iTunes	D-Calendar Free	Mark Ziemer	Birth Control Reminder
GP	Take it Off (Patch)	MobilAppSoftware	Birth Control Reminder
GP	Take it Out (Ring)	MobilAppSoftware	Birth Control Reminder
GP	Birth Control Pill	Ben Basha	Birth Control Reminder
Both	The Pill	Stephane Queraud	Birth Control Reminder
GP	Contraceptive Pill Alarm	Michael Asbeck	Birth Control Reminder
GP	Free Islamic Sex Education	Indonesian Brains Solution	Contraceptive Information
GP	Penis Condom	noobApps	Contraceptive Information
GP	Birth Control For Men	ConstantaSoft Inc	Contraceptive Information
iTunes	Sexual Positions Free for iPhone	iu Yue	Contraceptive Information
GP	Methods for Birth Control	T.Awadh	Contraceptive Information
Both	Trojan Real.good.sex	Church & Dwight Co. Inc	Contraceptive Information
GP	Condom Size	Consurgo	Contraceptive Information
iTunes	Illustrative Birth Control	Pratik Solanki	Contraceptive Information
iTunes	Pregnancy prevention, birth control techniques	Socl Solution	Contraceptive Information
iTunes	Birth Control	Lee Sunja	Contraceptive Information
GP	Virtual Nurse - Birth Control	Senstore - Powered by Harvard Medical School	Contraceptive Information
GP	How To Skip Pregnancy	Christian Wilkes	Contraceptive Information
iTunes	Safe Sex	ASD Soft	Contraceptive Information
iTunes	Mayo Clinic About Birth Control:	Mayo Clinic	Contraceptive Information

	Options for You		
GP	Get Ready	C-Dimension Ltd	Contraceptive Information
iTunes	Candies Cry Baby	York & Chapel	Educational game
iTunes	Condom Pro	Bedsider LLC	Educational game
Both	UnderControlCA	Victor Guana	Educational Game
GP	Girls Period 2	Zeromindlabs	Fertility Tracking
iTunes	Night Ovulation Calendar	Yauheni Shauchenka	Fertility Tracking
iTunes	Pink Ovulation Calendar	Yauheni Shauchenka	Fertility Tracking
iTunes	iGyno for Men	Mirco Bettelini	Fertility Tracking
iTunes	Monthly Cycles - Period Tracker	Deltaworks Limited	Fertility Tracking
iTunes	myPeriod Tracker	Emre Oencue	Fertility Tracking
iTunes	Period.Me - track your girlfriend period	Mladjan Antic	Fertility Tracking
iTunes	Safe Period Tracker	Phuong Tran Hoai	Fertility Tracking
iTunes	MyIntuition	Alfredo da Silva	Fertility Tracking
GP	Ovulation&Pregnancy Calendar	Enformativa	Fertility Tracking
iTunes	Period Tracker, Ovulation & Fertility	Lovetap	Fertility Tracking
iTunes	Ovulation calendar free - Conception & Pregnancy Calculator	Megakey Trans	Fertility Tracking
iTunes	i-femion	Nico Becherer	Fertility Tracking
iTunes	Lady Period	Viewscenes Corp	Fertility Tracking
iTunes	Safe Sex Calculator	jean Fages	Fertility Tracking
Both	Period Diary Supreme Lite	iBoxman Studio	Fertility Tracking
GP	Simple Period	beginsoft.kr	Fertility Tracking
GP	Fertility Diary - Maybe Baby	Tanu Kush	Fertility Tracking
GP	HappySexDate	gelato_cooper	Fertility Tracking
GP	Menstrual Fertility Calendar	z-mobile	Fertility Tracking
GP	Menstrual Diary	EONSOFT	Fertility Tracking
iTunes	A Safe Period Calculator	Tasnim Ahmed	Fertility Tracking
iTunes	Period Tracker	GP Apps	Fertility Tracking

	Lite		
iTunes	Safe Sex Period Checker	Ashfak Ahmed	Fertility Tracking
iTunes	Ovulation and Pregnancy Calendar	Vipos Web Apps	Fertility Tracking
iTunes	Groove - Period & Fertility Tracker	Groove, LLC	Fertility Tracking
iTunes	Period Tracker / Calendar Free App (My Period Tracker)	linchpin technologies Private limited	Fertility Tracking
Both	CycleProGo	The Couple to Couple League	Fertility Tracking
GP	Woman Calendar Lite. Menstrual	Krystian Kaminski	Fertility Tracking
GP	iPeriod Period Tracker	Winkpass Creations, Inc	Fertility Tracking
iTunes	Lily Cal (Period Tracker and Cycle Diary)	Whimsical Inc	Fertility Tracking
GP	Menstrual Cycle - Woman Log	4Rice Group	Fertility Tracking
GP	Period Calendar Free	Artin Studio	Fertility Tracking
GP	That Time of the Month Free	ToRo Software	Fertility Tracking
GP	Fertility Calendar	Msc	Fertility Tracking
GP	Period + Ovulation Calendar	Dynamic Woman	Fertility Tracking
Both	My Cycles Period and Ovulation	MedHelp, Inc - Top Health Apps	Fertility Tracking
iTunes	Pregnancy Due Date & Fertility Calculator	BabymedLLC	Fertility Tracking
iTunes	Period & Fertility Calendar	Pinch Swipe Tap Pty Ltd	Fertility Tracking
iTunes	NFP Manager: sympto	SymptoTherm Foundation	Fertility Tracking
GP	Month-Safe-SexDays:MC recorder	9 Soft Technologies Co.	Fertility Tracking
iTunes	2Day Method	Cycle Technologies	Fertility Tracking
iTunes	Cycles - Period & Fertility tracker	Perigee AG	Fertility Tracking
GP	OvuView:	sleekbit	Fertility Tracking

GP	Ovulation & Fertility BodyTemp and Period Monitor	Torchlight Innovation	Fertility Tracking
GP	Period View	Fertility Council	Fertility Tracking
GP	Magicday - Period Tracker	Goodoc	Fertility Tracking
iTunes	iGyno	Mirco Bettelini	Fertility Tracking
GP	WomanLog Calendar	Pro Active App	Fertility Tracking
iTunes	Ovulation Calendar Ladytimer Free	Vipos.com	Fertility Tracking
GP	My Period Tracker / Calendar	Linchpin Health	Fertility Tracking
GP	Period Tracker (Pink Pad)	Alt12 Apps	Fertility Tracking
GP	My Days - Period & Ovulation â„¢	Christian Albert Mueller	Fertility Tracking
iTunes	Pink Pad Period & Fertility Tracker	Alt12 Apps, LLC	Fertility Tracking
GP	My Days X	Christian Albert Mueller	Fertility Tracking
GP	Period Calendar / Tracker	ABISHKKING	Fertility Tracking
iTunes	iOvulation	Prolog Inc	Fertility Tracking
iTunes	Ovulation Lite	Dynadel	Fertility Tracking
GP	Intimity-Tracking my own cycle	Twinbo	Fertility Tracking
GP	Safe Love Calendar	Peix Software	Fertility Tracking
GP	New Love Calendar	myNanoEra	Fertility Tracking
Both	Kindara Fertility Tracker	Kindara, Inc.	Fertility Tracking
GP	Period Tracker / Calendar	yueyuejia.com	Fertility Tracking
GP	Woman's DIARY period cal	HighLab Co.,Ltd.	Fertility Tracking
Both	LoveCycles Menstrual Calendar	Plackal Tech	Fertility Tracking
iTunes	iCycleBeads Lite	Cycle Technologies	Fertility Tracking
GP	NaturalCycles fertility	NaturalCycles AG	Fertility Tracking
GP	Woman Calendar	SoftOrbits	Fertility Tracking
GP	CycleBeads Period & Ovulation	Cycle Technologies, Inc.	Fertility Tracking
GP	Lady Cycle	unidesigner	Fertility Tracking

GP	NFP sympto plus	Harri Wettstein	Fertility Tracking
Both	Glow Ovulation Period Tracker	Glow Inc	Fertility Tracking
iTunes	Pregnancy Test Checker Free	Inventive Mobile	Pregnancy Test
GP	Pregnancy Test Pro (2)	Horten	Pregnancy Test
GP	Pregnancy Test Pro	Vaddev	Pregnancy Test
GP	Pregnancy Test Dr Diagnozer	Extremedia	Pregnancy Test
GP	Pregnancy Test Lite	Jacek Miszczyk	Pregnancy Test
GP	Pregnancy Test	Aleksey Gubskiy	Pregnancy Test
GP	Free Pregnancy Test	Good Smile Studios	Pregnancy Test
iTunes	Pregnancy Test & Pregnancy Symptom Checker Quiz	Rebellion Media	Pregnancy Test
GP	Pregnancy Test & Symptom Quiz	Rebellion Media	Pregnancy Test
iTunes	Pregnancy Test Pro	Rebellion Media	Pregnancy Test
Both	Rachel House	mobile.Earth inc	Centers & Resources
GP	LoneStarFamilyHealth	AppManTx	Centers & Resources
GP	Jackson Healthcare for Women	bfac.com Apps	Centers & Resources
GP	AAA Pregnancy Resource Center	Xel Mobi Apps	Centers & Resources
iTunes	Alternatives	Black Eyed Apps, LLC	Centers & Resources
GP	Possibly Pregnant	Xel Mobi	Centers & Resources
iTunes	Parkgate Pregnancy Clinic	Mark Chase	Centers & Resources
GP	Pregnancy Help Center	Xel Mobi Apps	Centers & Resources
Both	Crossroads Pregnancy Center	Xel Mobi Apps	Centers & Resources
iTunes	ABC Women's Clinic	Mark Chase	Centers & Resources
Both	Safe Harbor	Xel Mobi Apps	Centers & Resources
Both	First Look	Xel Mobi Apps	Centers & Resources
Both	Birth Choice of Temecula	3 Cord Mark	Centers & Resources
iTunes	C.C.P.C.	Mark Chase	Centers & Resources
iTunes	Mid Cities	Mark Chase	Centers & Resources
Both	Planned	Planned	Centers & Resources

	Parenthood Care	Parenthood Federation of America	
iTunes	Open Arms Pregnancy Center	Matthew Flaig	Centers & Resources
iTunes	Options for Women NJ	Mark Chase	Centers & Resources
iTunes	PossiblyPreg	Mark Chase	Centers & Resources
iTunes	Pregnancy Resource Center	Mark Chase	Centers & Resources
iTunes	Real Options	Mark Chase	Centers & Resources
iTunes	Women's Resource Center of NE Wyoming	Mark Chase	Centers & Resources
iTunes	Door of Hope	Mark Chase	Centers & Resources
iTunes	AlphaCenter	Mark Chase	Centers & Resources
iTunes	Robbinsdale Women's Center	Mark Chase	Centers & Resources
iTunes	MyWC	Mark Chase	Centers & Resources
iTunes	New Hope Clinic	Mark Chase	Centers & Resources
iTunes	Sunrise Women's Clinic	Mark Chase	Centers & Resources
iTunes	TurningPoint	Mark Chase	Centers & Resources
iTunes	The Choice - it's kind of a big deal	Project Your Choice LLC	Centers & Resources
iTunes	Heart2Heart	Mark Chase	Centers & Resources
iTunes	PH Center	Mark Chase	Centers & Resources
iTunes	The Source.Org	Mark Chase	Centers & Resources
iTunes	Islands PCC	Mark Chase	Centers & Resources
iTunes	Hope Women's Centers	Mark Chase	Centers & Resources
iTunes	First Choice	Mark Chase	Centers & Resources
iTunes	Options Women's clinic	Mark Chase	Centers & Resources
iTunes	Conifer Sex Health	Thomas Benjamin Ltd	Centers & Resources
iTunes	Women's Health: Facts & Tips…	Michael Quach	SRH information
iTunes	Illustrative Menstrual Cycle and Sex	Pratik Solanki	SRH Information
GP	Reproductive Health	Keysha	SRH Information
iTunes	Safe Sex Tips	Dharmendra Solanki	SRH Information
iTunes	SmartSex	Pando Health Resources, LLC	SRH Information

iTunes	Safer Sex - not a dupe	Jo Langford	SRH Information
iTunes	PreConception Health Now	Kids Connected By Design	SRH information
GP	SexPositive	University of Oregon	SRH Information
GP	Find Help in Ireland	Glandore Systems	SRH Information
GP	Sex Education for Children	Fas F	SRH Information
GP	Healthy Sexual Life	Levant App	SRH Information
GP	SAFE	Amphibia	SRH Information
iTunes	SafeSex Guide	Mobile Identity Danmark ApS	SRH information
Both	Sexual Health Guide	GIRT Mobile	SRH Information
GP	No Worries	Smartphone Media/ Wiltshire College, Terrence Higgins Trust, & Salisbury PCT	SRH Information
Both	My Sex Doctor	MYSD LTD	SRH Information
GP	SexInfoOnline	SexInfoOnline	SRH Information
iTunes	iCondom	Morgane Danielou	SRH Service or Condom Locator
iTunes	iCondom Coventry	Raaza Ltd	SRH Service or Condom Locator
iTunes	iCondom Philly	Raaza Ltd	SRH Service or Condom Locator
iTunes	Wrapit	Julian Locke	SRH Service or Condom Locator
GP	Count and Find Condom	Andrea Rocco Lotronto	SRH Service or Condom Locator
GP	Condom Finder	CARES	SRH Service or Condom Locator
GP	Kent C Card	Kent Community Health NHS Trust	SRH Service or Condom Locator
GP	CaSH2U	ICE	SRH Service or Condom Locator
GP	NYC Condom	NYC Health	SRH Service or Condom Locator
iTunes	NYC Condom Finder	Jennifer Medina Matsuki	SRH Service or Condom Locator
iTunes	Sexual Health Liverpool	Glow New Media Ltd	SRH Service or Condom Locator
iTunes	fpa - Find a clinic	Family Planning Association	SRH Service or Condom Locator
GP	Health Squad	Mount Sinai Health Systems	Young Adult SRH Information

Both	Stand Up Girl	Neutrino Graphics	Young Adult SRH Information
GP	Mpower	Xel Mobi Apps	Young Adult SRH Information
GP	UCT Safe Sex	Information Systems Dept - University of Cape Town	Young Adult SRH Information
iTunes	The Real Deal	Core of Life	Young Adult SRH Information
GP	Safer Sex - not a dupe	C-Dimension Ltd	Young Adult SRH Information
GP	UCC Student Health	UCC Health	Young Adult SRH Information
iTunes	NeedTayKnow	Faff Digital	Young Adult SRH Information
Both	gPower	Georgia Campaign for Adolescent power & Potential	Young Adult SRH Information
Both	KIS-SK (Keep It Safe SK)	CollegeMobile, Inc	Young Adult SRH Information
Both	Teens in NYC	NYC Department of Health and Mental Hygiene	Young Adult SRH Information
GP	Girls Incorporated of Lynn	MDPH Office of Adolescent Health and Youth Development	Young Adult SRH Information
iTunes	SafeSex101	Associated Students UCLA	Young Adult SRH Information
iTunes	C&SH Somerset	Thomas Benjamin Ltd./ National Health Service	Young Adult SRH Information
GP	My Sex Doctor Lite	MYSD LTD; NHS	Young Adult SRH Information
iTunes	Your Choice Your Voice	National Health Service and Bromley Healthcare	Young Adult SRH Information

III. Grading Criteria for Contraceptives & Best Practices and Overall Scores

Domains	Points: Contraceptives & Best Practices	Points: Overall Score
**Pregnancy Prevention Best Practices (Taylor et al., 2011)**		
Screens or provides resources for intimate partner	1	1

violence		
Screens or provides resources for substance abuse	1	1
Inquires about life plan or family planning consideration	1	1
Provides contraceptive counseling including purpose or importance of family planning	1	1
Provides pregnancy test referral	1	1
Describes abortion options or services	1	1
Counsels on use of emergency contraception	1	1
	**7**	**7**
**Description of Contraceptive Methods & Clinical Services (Continued)**		
Provides description of any aspect of male condoms	1	1
Provides description of any aspect of female condoms	1	1
Provides description of any aspect of diaphragms	1	1
Provides description of any aspect of contraceptive sponge	1	1
Provides description of any aspect of cervical cap	1	1
Provides description of any aspect of oral contraceptives	1	1
Provides description of any aspect of contraceptive implant	1	1
Provides description of any aspect of intra-uterine device (IUDs)	1	1
Provides description of any aspect of the contraceptive patch	1	1
Provides description of any aspect of the contraceptive ring	1	1
Provides description of any aspect of spermicide	1	1
Provides description of any aspect of emergency contraception (EC)	1	1
Provides description of any aspect of injectable contraception	1	1
Provides description of any aspect of permanent contraception	1	1
	**14**	**14**
**Description of Contraceptive Methods & Clinical Services (Continued)**		
Describes confidentiality of clinical services		1
Describes cost of in-person services or notes that they are free		1
Describes cost of contraceptives or notes that they are free		1
Describes male condom effectiveness rate		1
Describes how to use male condoms		1
Describes female condom effectiveness rate		1

Describes how to use female condoms		1
Describes diaphragm effectiveness rate		1
Describes how to use diaphragm		1
Describes sponge effectiveness rate		1
Describes how to use sponge		1
Mentions cervical cap effectiveness rate		1
Mentions cervical cap insertion procedure		1
Mentions cervical cap length of effectiveness		1
Describes oral contraceptives effectiveness rate		1
Describes how to use oral contraceptives		1
Describes injectable contraception effectiveness rate		1
Describes how long injectable contraception is effective		1
Describes implant effectiveness rate		1
Describes how long the implant is effective		1
Describes IUD effectiveness rate		1
Describes how long the IUD is effective		1
Describes the patch effectiveness rate		1
Describes how to use the patch		1
Describes how long the patch is effective		1
Describes the ring effectiveness rate		1
Describes how to use the ring		1
Describes how long the ring is effective		1
Describes spermicide effectiveness rate		1
Describes how to use spermicide		1
Describes early withdrawal		1
Describes early withdrawal effectiveness rate		1
Describes when to withdraw		1
Describes permanent contraception effectiveness rate		1
Describes permanent contraception procedure		1
Describes fertility tracking		1
Describes fertility tracking effectiveness rate		1
Describes how fertility tracking prevents pregnancy		1
Describes EC effectiveness rate		1
Explicitly states that EC is not an abortion or will not terminate pregnancy		1
Information about dual protection		1
Information about where to get contraceptives		1
Information about contraceptive risks or side effects		1
Information about side effect management or switching		1

Information about sexually transmitted infections (STIs)		1
Information about STI testing		1
Notes that app is not a replacement for medical advice		1
Makes it clear to reviewers what steps to take to avoid unintended pregnancy (weighted by 10)		10
		57
**User Interface**		
Includes GPS		1
Includes maps navigation		1
Locates clinics or services near user		1
Locates contraceptives near user		1
Interactive (can set personal preferences or profile and get tailored feedback)		1
Supports appointment scheduling		1
Customizable look (skins/backgrounds)		1
Supports public communication (forum or can post to Facebook)		1
Supports direct communication (can chat, text or call from app)		1
Supports push notifications		1
Offers Main Menu or Navigation bar		1
Text and images are clear and legible		1
Offers movies/films		1
Offers audio		1
Offers tutorial on how to use the app		1
App functions as a decision-aid		1
		16
**Total Scores Possible**	**21**	**94**
